# Selection of Novel Reference Genes by RNA-Seq and Their Evaluation for Normalising Real-Time qPCR Expression Data of Anthocyanin-Related Genes in Lettuce and Wild Relatives

**DOI:** 10.3390/ijms24033052

**Published:** 2023-02-03

**Authors:** Inés Medina-Lozano, María Soledad Arnedo, Jérôme Grimplet, Aurora Díaz

**Affiliations:** 1 Department of Plant Sciences, Agrifood Research and Technology Centre of Aragon (CITA), Avd. Montañana 930, 50059 Zaragoza, Spain; 2 AgriFood Institute of Aragon–IA2, CITA-University of Zaragoza, 50013 Zaragoza, Spain; 3 Ramiro Arnedo S.A. Paraje La Molina 54, Las Norias de Daza, 04716 Almería, Spain

**Keywords:** BestKeeper, Delta Ct method, drought stress, geNorm, *Lactuca sativa* L., NormFinder, leaf colour, RefFinder, tissues, transcriptomics

## Abstract

Lettuce is a popular vegetable source of bioactive compounds, like anthocyanins, powerful antioxidants present in red and semi-red varieties. Selection of reliable reference genes (RGs) for the normalization of real-time quantitative PCR (qPCR) data is crucial to obtain accurate gene expression results. Among the genes with totally unrelated biological functions, six candidate RGs (*ADF2*, *CYB5*, *iPGAM*, *SCL13*, *TRXL3-3*, and *VHA-H)* with low variation in expression according to RNA-seq analyses, were selected for future expression studies of anthocyanin-related genes in three different experiments: leaf colour comparison (green vs. red) in commercial varieties; tissue comparison (leaf vs. stem) in a wild relative; and drought stress experiment in commercial and traditional varieties, and a wild relative. Expression profiles of the candidate RGs were obtained by qPCR and their stability was assessed by four different analytical tools, geNorm, NormFinder, BestKeeper, and Delta Ct method, all integrated in RefFinder. All results considered, we recommend *CYB5* to be used as RG for the leaf colour experiment and *TRXL3-3* for the tissue and drought stress ones, as they were the most stable genes in each case. RNA-seq is useful to preselect novel RGs although validation by qPCR is still advisable. These results provide helpful information for gene expression studies in *Lactuca* spp. under the described conditions.

## 1. Introduction

Lettuce (*Lactuca sativa* L.) is one of the most important leafy vegetables worldwide and its popularity is yearly increasing. Moreover, it is a valuable source of bioactive compounds [1], which have beneficial effects on human health. One of them are anthocyanins, water-soluble phenolic compounds with powerful antioxidant properties that help to prevent some human diseases, like cardiovascular and neuronal disorders, cancer or diabetes [2]. 

In lettuce, anthocyanins are responsible for the red colour, so they are exclusively present in red and semi-red varieties. In some previous studies, transcriptomic analyses have been carried out to compare green and red-leaf varieties [3,4,5] or to elucidate the accumulation of anthocyanins in lettuce [6,7]. There is a limited number of gene expression studies carried out in tissues different from leaves, like roots and stems, either in lettuce or its wild relatives. Among them, very few are focused on genes involved in the biosynthesis of bioactive compounds, like *LsHPPD* (4-hydroxyphenylpyruvate dioxygenase), a gene encoding for an enzyme from the pathway of vitamin E biosynthesis [8]. In this sense, lettuce wild relatives, e.g., *Lactuca serriola* L., have been used to explore the differential expression of candidate genes responsible for the synthesis of antioxidant compounds [7,9]. Transcriptomic studies to evaluate the response to different abiotic stresses, e.g., cold [10], heat [11,12], heavy metals [13,14], drought [15,16] and UV radiation [17] have been performed in lettuce. However, before this work, anthocyanin regulation has not been studied either under drought stress or in different tissues in the genus *Lactuca*. 

Real-time quantitative PCR (qPCR) is nowadays the most widely used technique for gene expression studies, due to its high specificity and sensitivity, as well as its immediacy in delivering results [18]. Nevertheless, a correct quantification of gene expression relies on an accurate normalisation of qPCR data that minimizes differences resulting from variation among samples and experimental errors. The most common normalisation method is the use of the so-called reference genes (RGs) as internal reaction controls. Ideally, RGs have to exhibit a relatively constant expression regardless of the nature of the samples and the experimental conditions [19].

Traditionally, RGs have been selected among those considered as housekeeping genes, since they encode proteins implied in essential cellular processes and then, they are expected to show stable expression throughout the different tissues and conditions. Some typical examples of housekeeping genes used as RGs in plants are actin (*Act*), glyceraldehyde 3-phosphate dehydrogenase (*GAPDH*), elongation factor 1-α (*EEF1-α*), ubiquitin (*Ubq*), tubulin (*Tub*), and 18S ribosomal RNA (*18SrRNA*) [20,21,22,23]. However, the use of conventional housekeeping genes as RGs is not always appropriate. This has been demonstrated in plants by many studies in which these genes show notable expression variation depending on the tissue, the physiological state, the developmental stage, and the experimental conditions, as reviewed in [19,24,25]. That is, there are no genes whose expression is stable in all possible materials (cells and tissues) and under any conditions assayed. So, for each experimental design, it will be necessary to test the available genes and, if they show variable expression, to search for new genes and verify that they are suitable as RGs.

In lettuce, novel candidate RGs to study gene expression variation throughout the day and at different developmental stages [26] or in response to different abiotic stresses [27] have been tested. The selection was based on genes that had been previously identified as stable in other species. Nevertheless, thanks to advances in next generation sequencing technologies, it is more and more common to use RNA-seq data as a source of novel candidate RGs, as it has already been done in grapevine, soybean and apple [28,29,30]. 

In the present study, stability of six candidate RGs has been evaluated in order to identify suitable genes for the expression analyses of genes related to anthocyanin accumulation in lettuce and wild relatives. Their stability has been analysed in three different experiments: comparison of leaf colour in green and red commercial varieties of lettuce, comparison of tissues (leaf and stem) in a wild relative species, and response to drought stress in cultivated lettuce (commercial and traditional varieties) and in a wild relative species. To the best of our knowledge, this is the first time that the selection of candidate RGs in lettuce has been based on RNA-seq data. The gene expression analyses have not only been carried out in lettuce commercial varieties, but they have also been extended to plant material scarcely studied before [1], e.g., two wild relatives (*Lactuca* spp.) and one lettuce traditional variety.

## 2. Results

### 2.1. Selection of Candidate reference genes (RGs) Based on RNA-seq Data

A total of six candidate RGs (Table 1, Table S1) were selected from RNA-seq analyses carried out in *Lactuca* spp. in three different experiments (see “Materials and Methods” section), attending to different selection criteria because classic housekeeping genes previously used in plants did not show stable expression across the different conditions (Table 2). The first one was the expression stability: values of log_2_FC (fold change) ranged from −0.75 to 1.03 (Table S2). Then, among those genes that were common in the three experiments, genes with a wide sequence coverage were selected. Finally, one-way analysis of variance (ANOVA) and Student’s *t*-test for mean comparison were carried out for gene count data among the groups of each experiment, and six genes without significant differences (unlike the six classic housekeeping genes analysed) were selected (Table 2). Gene function was also taken into account; five out of six RGs had a structural or metabolic function: cellular component (*ADF2*), ion transport (*CYB5* and *VHA-H*), metabolism (*iPGAM*), and photosynthesis (*TRXL3-3*). However, a probable transcription factor (*SCL13*) was also included, as it is required for hypocotyl elongation regulation during de-etiolation and, therefore, it is not related to anthocyanin content regulation.

Some of the RGs that have been traditionally used, such as actin, tubulin, ubiquitin, etc., were planned to be included in this study, but they did not meet all the selection criteria previously described for at least one of the experiments (Table 2).

### 2.2. Expression Profile of Candidate RGs

**Figure 1:**
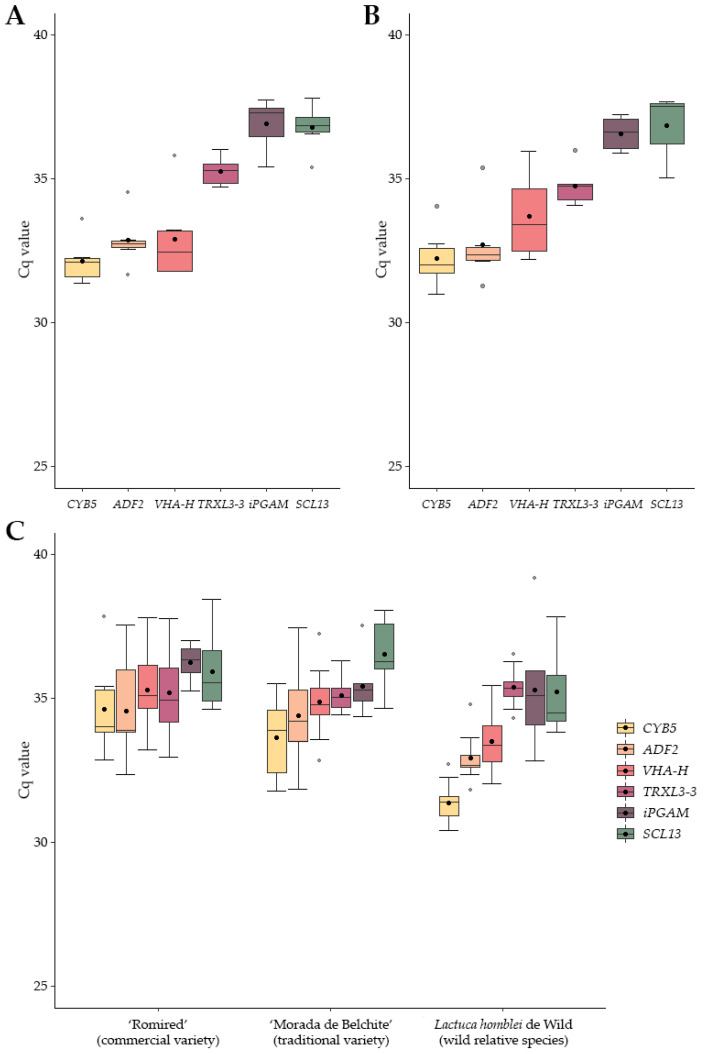
Expression levels, Cq (quantification cycle) values assessed by real-time qPCR (quantitative PCR), of six tested candidate RGs across the samples in three experiments: (**A**) comparison of leaf colour in green and red lettuce commercial varieties; (**B**) comparison of tissues (leaf and stem) in a wild relative species; and (**C**) drought stress in a commercial variety, a traditional variety, and a wild relative species. Lower and upper ends of the boxes represent the 25th and 75th percentiles, respectively, and whisker caps indicate the minimum and maximum values. Horizontal bars and black and grey dots depict the median, mean and outliers, respectively.

Expression levels of the candidate RGs were determined by real-time qPCR using Cq (quantification cycle) data, also referred as Ct (threshold cycle). Cq values of the RGs ranged from 31.38 to 37.82 in green vs. red, from 30.99 to 37.67 in leaf vs. stem, and from 30.43 to 39.17 in the drought stress experiments (Figure 1 and Table S3). *CYB5* and *ADF2* showed the highest expression levels (lowest mean Cq values) in the three experiments, whereas the genes with the lowest expression levels (highest mean Cq values) were *iPGAM* and *SCL13*, except for the wild relative species in the drought stress experiment, that was *TRXL3-3* (followed by those same two).

The variation width of Cq values across the samples (without considering the outliers) was very different for the six candidate RGs within each experiment (Figure 1). Gene showing the narrowest variation was *ADF2*, except for the drought stress experiment in the cultivated varieties, in which happened to be *iPGAM* (Figure 1 and Table S3). Nevertheless, a low variation of Cq values is essential but not sufficient to guarantee the stability of RGs expression.

### 2.3. Analysis of Gene Expression Stability in Accessions of Lactuca: Different Leaf Colour, Tissues, and Drought Stress Conditions

Expression stability of the six candidate RGs in the previously described experiments was assessed by four different analytical tools: geNorm [31], NormFinder [32], and BestKeeper [33] algorithms, and the Delta Ct (ΔCt) method [34], all included in the RefFinder software [35]. M (in geNorm) and SV (stability value, in NormFinder, BestKeeper, and ΔCt) measured the relative expression stability, such that lower M and SV indicated a more stable expression. Nevertheless, each method of analysis follows different statistical procedures (explained in Section 4.5), and the input data are also different, as geNorm and NormFinder algorithms use Cq data corrected with the efficiency values (CqE), whereas BestKeeper and the ΔCt method use mean Cq data. Therefore, stability rankings for each gene within the three experiments show slight differences according to the analytical method applied, as shown in Table 3.

**Figure 2:**
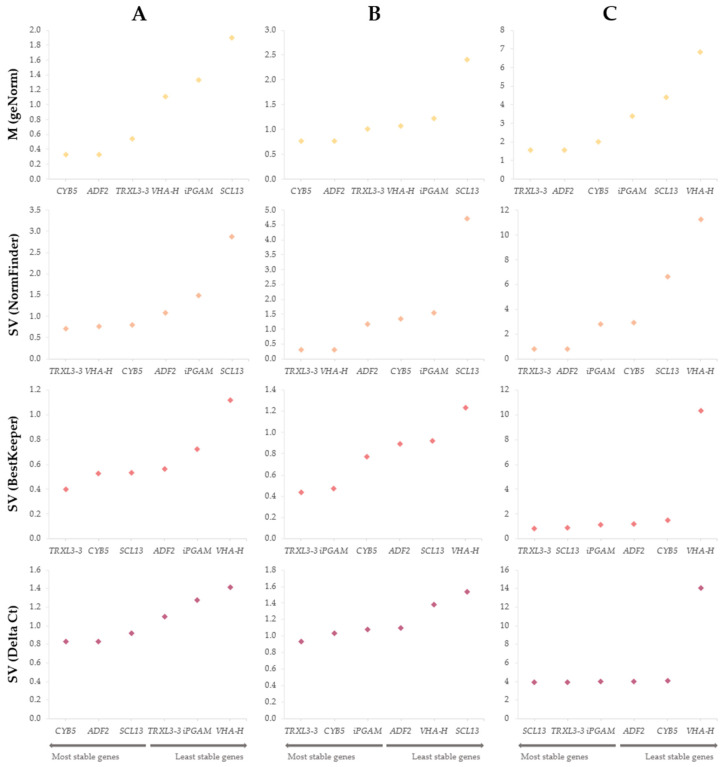
Expression stability of six tested candidate reference genes (RGs) calculated by (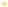) geNorm (M), (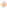) NormFinder, (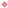) BestKeeper, and (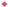) ΔCt (SV) methods in three experiments: (**A**) comparison of leaf colour (green and red) in lettuce commercial varieties; (**B**) comparison of tissues (leaf and stem) in a wild relative species; and (**C**) under drought stress in a commercial variety, a traditional variety, and a wild relative. The most stable genes are represented on the left and the least stable on the right of the graph.

For the comparison of gene expression in green vs. red leaf colour (Figure 2A and Table 3), the two most stable genes were *CYB5* and *ADF2*, *TRXL3-3* and *VHA-H*, *TRXL3-3* and *CYB5*, and *CYB5* and *ADF2* according to geNorm, NormFinder, BestKeeper and ΔCt, respectively. By contrast, the gene with the lowest stability value was *SCL13* according to geNorm and NormFinder, and *VHA-H* according to BestKeeper and ΔCt, being *iPGAM* found the second least stable gene with the four analytical tools.

When comparing leaf vs. stem tissues (Figure 2B and Table 3), the top two positions in the ranking were occupied by *CYB5* and *ADF2*, *TRXL3-3* and *VHA-H*, *TRXL3-3* and *iPGAM*, and *TRXL3-3* and *CYB5*, applying geNorm, NormFinder, BestKeeper and the ΔCt method, respectively. In contrast, *SCL13* was ranked at the bottom according to geNorm, NormFinder and ΔCt, whereas *VHA-H* was the most unstable gene according to BestKeeper. 

Finally, in the drought stress experiment (Figure 2C and Table 3), the most stable genes resulted to be *TRXL3-3* and *ADF2,* according to geNorm and NormFinder, and *TRXL3-3* and *SCL13* (though in reverse order), according to BestKeeper and ΔCt. In this case, *VHA-H* showed the lowest expression stability according to the four methods of analysis. 

Due to the slight differences observed when using the different analytical tools, a comprehensive ranking that integrates stability values calculated with all of them was created for each experiment (Table 4), following the procedure described in Section 4.5. Thus, the most stable RG for green vs. red was *CYB5* followed by *TRXL3-3*. These two genes reached the two top positions again in the leaf vs. stem experiment, although in the opposite order. Finally, *TRXL3-3* was also found to be the best RG for the drought stress experiment followed, in this case, by *ADF2*. On the other hand, *iPGAM*, *SCL13*, and *VHA-H* resulted to be the most unstable genes for green vs. red, leaf vs. stem, and drought stress experiments, respectively. 

Two more comprehensive rankings were created considering separately analytical methods that use either CqE (geNorm and NormFinder) or Cq (BestKeeper and ΔCt) data (Table S4). In comparison with the ranking that comprises results from the four analytical tools (Table 4), the genes at the highest positions coincided, except in the case of the BestKeeper + ΔCt ranking for the drought stress experiment. Similarly, the genes in the lowest positions were mostly the same in the case of geNorm + Normfinder ranking (in reverse order in the leaf colour experiment) whereas they were slightly different for the BestKeeper + ΔCt ranking in two out of the three experiments (leaf colour and tissues).

## 3. Discussion

Robust normalization of real-time qPCR data by RGs is essential to obtain reliable results in gene expression studies. Furthermore, it is well known that identification of stable RGs is necessary for each experimental context. In this work, the suitability of six candidate RGs has been evaluated in expression studies of anthocyanin-related genes in three different experiments: comparison of leaf colour (green and red) in lettuce commercial varieties, comparison of tissues (leaf and stem) in a wild relative, and drought stress in two lettuce varieties (one commercial and one traditional) and a wild relative. 

Expression of genes with an effect on anthocyanin content has been scarcely studied in lettuce, except in red-leaf varieties in normal conditions [3,4,5,6,7]. The first step in this kind of analyses should be the selection of suitable RGs. Identification of stable genes as candidate RGs in lettuce has been previously tackled to assess gene expression throughout the day and in different developmental stages [26], and in the response to abiotic stresses [27]. Although RGs for the normalization of expression data under drought stress have already been identified, the target gene tested to validate the selected RGs was not directly related to anthocyanin accumulation [27]. In addition, RGs for the other two experiments (green vs. red and leaf vs. stem) and the plant material studied here have not been tested before, as far as we know. Furthermore, unlike in these previous studies in lettuce, selection of candidate RGs in this work was based on RNA-seq data. Through the bioinformatic analysis of these RNA-seq data, we selected a set of candidate RGs (*ADF2*, *CYB5*, *iPGAM*, *TRXL3-3*, *SCL13*, and *VHA-H*) that were different from those tested in [26,27]. Classic housekeeping genes used in plants [20,21,22,23] were discarded since in general they did not meet all our selection criteria (i.e., they were not stably expressed or the sequence coverage was insufficient or ANOVA F-value < 0.05) in at least one of the experiments. Therefore, it is the first time that this set of candidate RGs is tested in lettuce, to the best of our knowledge.

Four different analytical tools have been used to assess the six candidate RG stability and then a comprehensive ranking was elaborated to integrate the results from the four methods. According to this ranking, the two most stable genes, that is, the most suitable as RGs, were *CYB5* and *TRXL3-3* for green vs. red experiment, again *CYB5* and *TRXL3-3* for the leaf vs. stem experiment, and *TRXL3-3* followed by *ADF2* for the drought stress experiment (Table 4). In contrast, the most unstable genes and therefore, the less suited to be used as RGs, were *iPGAM*, *SCL13,* and *VHA-H*, for each of the three experiments above mentioned, respectively (Table 4). 

Most of the tested candidate RGs have a structural or metabolic function, except for *SCL13*, a probable transcription factor (Table 1). Even so, *SCL13* was selected because firstly, it revealed itself stable according to the RNA-seq results, and secondly, it participates in the regulation of the hypocotyl elongation during the seedling de-etiolation, that is a totally different process from those of our interest (anthocyanin biosynthesis/degradation and transport). However, in general, it exhibited a low position in the comprehensive stability ranking in our experiments (Table 4). In spite of being a regulatory element, its low stability might be due to the qPCR efficiency values obtained (Table S3) and not to its function as a transcription factor. This is supported by the fact that *SCL13* positions were generally higher in the rankings that did not take into account efficiency data (they used directly mean Cq values) and, hence, they were lower when efficiency data were considered (rankings that used CqE values) (Table 3). This happened for the three experiments, but the differences especially stood out in the drought stress experiment, where *SCL13* position dropped from the first in Bestkeeper + ΔCt ranking to the third in geNorm + NormFinder and the global rankings (Table 4 and Table S4). That is, correction with efficiency values makes *SCL13* become a less stable gene.

It is known that results of gene expression assays are hugely influenced by qPCR efficiency, and in turn, qPCR efficiency is very dependent on the primer design [36]. In our study, some efficiency values were relatively low (Table S3) as there were important sequence limitations during the primer design (Table S1). The two wild relative species included in this study, *Lactuca homblei* de Wild and *Lactuca squarrosa* (Thunb.) Miq., belong to the tertiary lettuce gene pool [37], so they are genetically distant from the cultivated lettuce, *L. sativa*. In the case of the drought stress experiment, the use of the same pairs of primers for *L. sativa* varieties and *L. homblei* was mandatory, whereas in the tissue experiment, different primers could have been designed though we opted for using the same to be able to establish comparisons among all the experiments, assuming some penalization in terms of efficiency. Therefore, a redesign of primers in a study without our limitations would probably improve the efficiency values and increase the expression stability of the candidate RGs that were negatively affected by it.

The rankings obtained using CqE data (geNorm + NormFinder) are more similar to the global one (Table 4) than those generated with Cq values (BestKeeper + ΔCt) (Table S4). This supports the importance of considering efficiency data, as it has already been reported in other crops like wheat [38]. So, we recommend the use of geNorm and NormFinder algorithms over BestKeeper and the ΔCt method. Indeed, in the last few years, geNorm and NormFinder seems to be the most frequently used tools to measure the RG stability in gene expression studies, especially in detriment of the ΔCt method. Nevertheless, in our study, differences among the three rankings are not important since those RGs identified as the most or least stable occupied generally the upper or lower positions, respectively (Table 4 and Table S4).

## 4. Materials and Methods

### 4.1. Plant Material and Experimental Designs

Three different experiments were performed using especially appropriate plant materials in each case (Table 5). First, to compare leaf colour, the commercial varieties ‘Begoña’ and ‘Romired’, green and red lettuces, respectively, were cultivated in winter 2018/2019. Second, to study different tissues (leaf and stem), a wild relative species with bushy growth habit, *L. squarrosa*, was grown in winter 2020/2021. Third, a drought stress experiment was carried out with the commercial variety ‘Romired’, the traditional variety ‘Morada de Belchite’, and the wild relative species *L. homblei*, cultivated in winter 2020/2021. Concretely, this experiment consisted of three different irrigation schedulings applied three weeks before harvesting: C (control or full irrigation, week 1: 1350 mL, weeks 2–3: 2100 mL/each), DI-1 (Deficit Irrigation 1, week 1: 450 mL, weeks 2–3: 150 mL/each), and DI-2 (weeks 1–3: 0 mL). In the leaf colour experiment, 3 plants per accession were cultivated in a greenhouse at Ramiro Arnedo S.A. (Almería, Spain). In the tissue experiment, 3 plants per accession were cultivated in a greenhouse at Agrifood Research and Technology Centre of Aragón (CITA, Zaragoza, Spain). In the drought stress experiment, 3 plants per accession for each of the 3 treatments (9 plants per accession in total) were cultivated in a greenhouse at CITA. In all cases, the plants were distributed following a complete randomized block design. Plants were grown in pots (30 × 25 cm and 11.7 L volume) with a mix of black and blonde peat (1:1) with fertilizer incorporated but without supplemental lighting to avoid the stimulation of anthocyanin synthesis. After a period ranging from 3 (2018/2019) to 3 and a half months (2020/2021), two leaves per plant (one inner and one outer) were collected, as well as parts of the stem in the case of *L. squarrosa*, so that the whole plant was represented. The samples were immediately frozen with liquid nitrogen and then kept at −80 °C until their lyophilization.

### 4.2. RNA Extraction and RNA-Seq Analysis

Total RNA was extracted from lyophilized samples using the NZY Total RNA Isolation kit (NZYtech Lda.-Genes and Enzymes, Lisbon, Portugal) and then treated with DNase to ensure elimination of DNA using the TURBO DNA-free^TM^ kit (Invitrogen, Waltham, MA, USA), according to manufacturers’ instructions. Quantity and purity of extracted RNA were measured with the Eukaryotic Total RNA Nanobioanalyzer Assay in a 2100 Bioanalyzer (Agilent Technologies, Santa Clara, CA, USA). The obtained RNA samples were used to build a total of 39 cDNA libraries resulting from the 3 experiments (leaf colour: 2 accessions × 3 replicates; plant tissue: 2 tissues × 3 replicates; drought stress: 3 accessions × 3 replicates × 3 irrigation regimes). All libraries were sequenced in both directions using the TruSeq Stranded mRNA protocol (Illumina, San Diego, CA, USA) with a NovaSeq 6000 S1 instrument (Illumina) to obtain from 32 to 111 million strand-specific pair-end reads of 100 base pairs (bp) each. Sequencing was performed at the National Centre for Genomic Regulation (CNAG-CRG, Barcelona, Spain). 

Sequence analyses were performed using the Galaxy tool [39]. Adapter sequences were removed using Trimmomatic (Galaxy Version 0.38.1) [40] and reads were aligned to the lettuce reference genome Last_Salinas_V7 (GCF_002870075.2) using HISAT2 (Galaxy Version 2.2.1+galaxy0) [41], with a maximum length of 20,000 bp between exons. MarkDuplicates (Galaxy Version 2.18.2.2) and FixMateInformation (Galaxy Version 2.18.2.1) Picard tools [42] were used to filter out optical duplicates and to confirm mate-pairs, respectively. Read counts were generated using featureCounts (Galaxy Version 2.0.1+galaxy2) [43] and differential expression analyses were performed using edgeR (Galaxy Version 3.36.0+galaxy0) [44].

### 4.3. Selection of Candidate RGs

RNA-seq data were used to select the candidate RGs among those that met the criteria explained bellow. Firstly, all genes must show stable expression across accessions, tissues, or treatments within each assay. So, they were filtered for low values of the FC (fold change, log_2_(FC) ≤ 1) as values close to zero indicate that there is neither an increase nor a decrease in the gene expression among the groups compared, and the FDR (False Discovery Rate) threshold of 5% (adjusted *p*-value via the Benjamini and Hochberg method using the edgeR package [44]). Secondly, genes that presented a wide sequence coverage were selected. Finally, one-way analysis of variance (ANOVA) and Student’s *t*-test for mean comparison were performed for the gene counts among the groups within each experiment with JMP v5.1.2 software for Windows (SAS Institute Inc., Cary, NC, USA). Eventually, taking also into account their biological functions, six genes without significant differences were chosen: *ADF2*, *CYB5*, *iPGAM*, *SCL13*, *TRXL3-3*, and *VHA-H* (Table 1). They were mainly genes involved in constitutive processes, as they are expected to have stable expression in any tissue and under any circumstances. Specifically, they are genes encoding for cytoskeleton components (*ADF2*), enzymes catalysing metabolic reactions (*iPGAM* and *TRXL3-3*), and electron (*CYB5*) or proton (*VHA-H*) transporters. However, a probable transcriptional regulator of seed germination (*SCL13*) was also included with the intention of testing whether regulatory elements could be used as RGs provided that they do not participate in the same processes under study.

### 4.4. mRNA Isolation, cDNA Synthesis and Real-Time qPCR

mRNA purification from total RNA samples described above was performed using the Dynabeads mRNA DIRECT^TM^ kit (Invitrogen), followed by the cDNA synthesis using the NZY M-MuLV First-Strand cDNA Synthesis separate oligos kit (NZYTech), as recommended by manufacturers. Specific pairs of primers were designed for each selected candidate RG (Table 1) using OLIGO software version 6.45 (Cascade, CO, USA) and a consensus sequence from the three species under study as template, the cultivated lettuce (*L. sativa*), and the two wild relatives (*L. homblei* and *L. squarrosa*) were selected, paying special attention to exclude any ambiguity in their sequences. Real-time qPCR reactions were performed on a StepOnePlus^TM^ System (Applied Biosystems, Waltham, MA, USA). Each reaction was run in a final volume of 12 µL containing 1 µL of 1:40 diluted cDNA, 0.40 mM of forward and reverse primers (Integrated DNA Technologies, IDT, Coralville, Iowa, USA), and 1x NZYSupreme qPCR Green Master Mix, ROX plus (NZYTech). The amplification conditions were the following: 2 min at 95 °C and 40 cycles of 5 s at 95 °C, 15 s at 56–62 °C (Table 1) and 30 s at 72 °C. Melting curve analyses were carried out to verify the specificity of each reaction and it ranged from 72 °C to 95 °C with 0.3 °C increment per cycle. Two technical replicates per sample were performed and non-template controls were added to ensure that contamination with genomic DNA had not occurred. 

### 4.5. Stability Analysis of RGs

To evaluate gene expression stability of the set of RGs, the data were analysed with geNorm [31], NormFinder [32], and BestKeeper [33] algorithms, and the comparative ΔCt method [34], all of them integrated in RefFinder software [35]. geNorm algorithm calculates the expression stability value (M value) based on the average pairwise variation (V_n/n+1_) for a candidate RG with all the other tested genes. Lower M values indicate more stable expression. NormFinder uses an ANOVA-based model that calculates SV considering intra- and inter-group variation. Lower values represent more stability. BestKeeper ranks the stability of candidate RGs considering three variables: standard deviation (SD), coefficient of variation (CV) and correlation coefficient (r). The lower values of SD and CV and the higher values of r, the more stable is the gene expression (lower SV). The comparative ΔCt method first obtains the difference between the Cq of the treated and control samples. Then, it displays pairwise comparisons between the genes by calculating the mean SD. Lower values of SD, and hence of SV, indicate higher stability of the genes. For BestKeeper and ΔCt, mean values of Cq were used, whereas for geNorm and NormFinder, corrected values of Cq with the efficiency data were used, according to the formula CqE = Cq*(log(E)/log(2)) [45]. In all cases, mean data of two technical replicates per sample were used. 

Finally, a comprehensive ranking that comprises results from the four analytical tools (geNorm, NormFinder, BestKeeper, and the comparative ΔCt method) was elaborated for each of the three experiments. Firstly, individual rankings were created by assigning a certain weight to each gene according to the results obtained from each of these statistical methods. Then, a final weighted list was obtained by calculating geometric means from the four individual rankings for each RG. In addition, two more comprehensive rankings for each experiment were obtained following the same procedure, one comprising stability data from geNorm and NormFinder, and the other from BestKeeper and ΔCt, methods that use corrected and non-corrected values of Cq, respectively.

## 5. Conclusions

In this study, a novel set of six candidate RGs with low variation in expression levels in *Lactuca* spp. were selected based on an RNA-seq data analysis. Their RNA-seq expression was validated by qPCR and candidate RGs were subjected to analyses of expression stability through different analytical tools. We have found stable genes, hence suitable RGs for an accurate real-time qPCR normalization in three different experiments in the genus *Lactuca*. For studying the expression of anthocyanin-related genes in these *Lactuca* spp., we concretely recommend *CYB5* for the experiment comparing the green and red-leaf commercial varieties of lettuce; and *TRXL3-3* for the comparison of tissues (leaf and stem) in a wild relative species with bushy growth, and for the drought stress experiment in two cultivated varieties of lettuce (one commercial and one traditional) and in a wild relative. On the other hand, *iPGAM*, *SCL13*, and *VHA-H* should be avoided in all three experiments set out here, although an increase in efficiency values by primer redesign (when possible) might improve expression stability in some cases. Furthermore, the six candidate RGs studied here, but especially the two revealed as the most stable (*CYB5* and *TRXL3-3*), could be tested to verify if they show stable expression in any *Lactuca* spp. as long as the processes under study are not related to those aforementioned in which these six genes participate. The results of this study provide valuable information for future gene expression studies in different accessions of lettuce, different tissues, and conditions of stress. 

In addition, these stable RGs will be used as internal controls in future research on expression of genes related to anthocyanin content, among which we expect to find biosynthesis genes, genes encoding proteins of transport and transcription factors within each experiment: comparison of leaf colour, of tissues, and drought stress, respectively.

## Figures and Tables

**Table 1:** Description, primer sequences, amplicon length, and qPCR annealing temperature of six candidate reference genes (RGs).

Name	Description	Primer Sequence (5′–3′)	Amplicon Length (bp)	Annealing Temperature (°C)
*ADF2*	Actin-depolymerizing factor 2	F-TTGGAGAACCAGCAGAAAC	199	62
		R-CCATCAAGCTCTCTCTTGAAC		
*CYB5*	Cytochrome B5	F-GCACGCTACGAAAGAGG	80	59
		R-CAGGATGATCATCTAGAAAAGG		
*iPGAM*	Probable 2,3-bisphosphoglycerate-independent phosphoglycerate mutase	F-GGGAGATGTTTCAATTCCAAG	162	62
		R-CCCATTAGAGAAAGATGAGCAG		
*SCL13*	Scarecrow-like protein 13	F-AGTCGGTTAGCACGGTTA	100	56
		R-TTCGTGTTCGATTCTTGTT		
*TRXL3-3*	Thioredoxin-like protein 3-3	F-TGGTGTCGTGTTTGTGCAGAG	112	62
		R-GTTGGGTTGTTTCTGGGCATT		
*VHA-H*	V-type proton ATPase subunit H	F-TGCAAGTGATGATGTTTTGA	152	59
		R-TGCTTGAACAAATGAAGACC		

**Table 2:** F-values resulting from ANOVA of gene counts for traditional housekeeping genes and new candidate reference genes (RGs) among groups of each experiment: green vs. red, leaf vs. stem, and drought stress.

Gene ^a^	Green vs. Red	Leaf vs. Stem	Drought Stress		
			‘Romired’	‘Morada de Belchite’	*L. homblei*
*ACT*	0.564 ^ns^	0.938 ^ns^	0.421 ^ns^	0.018 *	0.036 *
*α-TUB*	0.635 ^ns^	0.001 **	0.435 ^ns^	0.637 ^ns^	0.643 ^ns^
*EEF1-α*	0.019 *	0.066 ^ns^	0.976 ^ns^	0.089 ^ns^	0.696 ^ns^
*GAPDH-2C*	0.028 *	0.027 *	0.407 ^ns^	0.302 ^ns^	0.593 ^ns^
*UBC32*	0.159 ^ns^	0.159 ^ns^	0.398 ^ns^	0.276 ^ns^	0.010 *
*UPL6*	0.471 ^ns^	0.013 *	0.003 **	0.013 *	0.168 ^ns^
*ADF2*	0.569 ^ns^	0.884 ^ns^	0.400 ^ns^	0.660 ^ns^	0.547 ^ns^
*CYB5*	0.449 ^ns^	0.270 ^ns^	0.723 ^ns^	0.371 ^ns^	0.673 ^ns^
*iPGAM*	0.833 ^ns^	0.773 ^ns^	0.128 ^ns^	0.418 ^ns^	0.210 ^ns^
*SCL13*	0.408 ^ns^	0.902 ^ns^	0.883 ^ns^	0.427 ^ns^	0.239 ^ns^
*TRXL3-3*	0.487 ^ns^	0.406 ^ns^	0.843 ^ns^	0.161 ^ns^	0.470 ^ns^
*VHA-H*	0.198 ^ns^	0.576 ^ns^	0.116 ^ns^	0.195 ^ns^	0.741 ^ns^

^a^*ACT*: actin; *α-TUB*: tubulin alpha chain; *EEF1-α*: elongation factor 1-alpha; *GAPDH-2C*: glyceraldehyde-3-phosphate dehydrogenase 2C; *UBC32*: ubiquitin-conjugating enzyme E2 32; *UPL6*: E3 ubiquitin-protein ligase UPL6. ns: non-significant; * *p* < 0.05; ** *p* < 0.01

**Table 3:** Stability values of each candidate reference gene (RG) obtained with geNorm (M), NormFinder, and BestKeeper algorithms, and the Delta Ct method (SV) in three different experiments: green vs. red, leaf vs. stem, and drought stress.

		geNorm		NormFinder		BestKeeper		Delta Ct	
Experiment	Ranking	Gene	M	Gene	SV	Gene	SV	Gene	SV
Green vs. red	1	*CYB5*	0.33	*TRXL3-3*	0.71	*TRXL3-3*	0.40	*CYB5*	0.83
	2	*ADF2*	0.33	*VHA-H*	0.77	*CYB5*	0.53	*ADF2*	0.83
	3	*TRXL3-3*	0.54	*CYB5*	0.81	*SCL13*	0.53	*SCL13*	0.92
	4	*VHA-H*	1.11	*ADF2*	1.09	*ADF2*	0.57	*TRXL3-3*	1.10
	5	*iPGAM*	1.33	*iPGAM*	1.49	*iPGAM*	0.72	*iPGAM*	1.28
	6	*SCL13*	1.90	*SCL13*	2.88	*VHA-H*	1.12	*VHA-H*	1.41
Leaf vs. stem	1	*CYB5*	0.77	*TRXL3-3*	0.30	*TRXL3-3*	0.44	*TRXL3-3*	0.93
	2	*ADF2*	0.77	*VHA-H*	0.30	*iPGAM*	0.47	*CYB5*	1.03
	3	*TRXL3-3*	1.01	*ADF2*	1.18	*CYB5*	0.77	*iPGAM*	1.08
	4	*VHA-H*	1.07	*CYB5*	1.34	*ADF2*	0.89	*ADF2*	1.10
	5	*iPGAM*	1.22	*iPGAM*	1.55	*SCL13*	0.92	*VHA-H*	1.38
	6	*SCL13*	2.41	*SCL13*	4.73	*VHA-H*	1.23	*SCL13*	1.54
Drought stress	1	*TRXL3-3*	1.54	*TRXL3-3*	0.77	*TRXL3-3*	0.85	*SCL13*	3.89
	2	*ADF2*	1.54	*ADF2*	0.77	*SCL13*	0.92	*TRXL3-3*	3.90
	3	*CYB5*	1.98	*iPGAM*	2.81	*iPGAM*	1.11	*iPGAM*	4.01
	4	*iPGAM*	3.40	*CYB5*	2.91	*ADF2*	1.23	*ADF2*	4.04
	5	*SCL13*	4.41	*SCL13*	6.62	*CYB5*	1.50	*CYB5*	4.07
	6	*VHA-H*	6.82	*VHA-H*	11.27	*VHA-H*	10.35	*VHA-H*	14.08

**Table 4:** Comprehensive stability ranking of six candidate reference genes (RGs) for three different experiments: green vs. red, leaf vs. stem, and drought stress.

Ranking	Green vs. Red	Leaf vs. Stem	Drought Stress
1	*CYB5*	*TRXL3-3*	*TRXL3-3*
2	*TRXL3-3*	*CYB5*	*ADF2*
3	*ADF2*	*ADF2*	*SCL13*
4	*VHA-H*	*VHA-H*	*iPGAM*
5	*SCL13*	*iPGAM*	*CYB5*
6	*iPGAM*	*SCL13*	*VHA-H*

**Table 5:** Plant material used in the three experiments of the present study.

Experiment	Accession Name	Species	Group	Leaf Colour	Year	Source ^a^	Accession Number
Leaf colour (green vs. red)	‘Begoña’	*Lactuca sativa* L.	Commercial variety	Green	2018/2019	Ramiro Arnedo Semillas S.A.	-
	‘Romired’	*Lactuca sativa* L.	Commercial variety	Red		CGN	CGN24713
Tissue (leaf vs. stem)	*Lactuca squarrosa*	*Lactuca squarrosa* (Thunb.) Miq.	Wild crop relative	Semi-red (red stems)	2020/2021	BGHZ	BGHZ5124
Drought stress (C vs. DI-1 vs. DI-2) ^b^	‘Romired’	*Lactuca sativa* L.	Commercial variety	Red	2020/2021	CGN	CGN24713
	‘Morada de Belchite’	*Lactuca sativa* L.	Traditional variety	Semi-red		BGHZ	BGHZ0527
	*Lactuca homblei*	*Lactuca homblei* De Wild	Wild crop relative	Semi-red		BGHZ	BGHZ5322

^a^ BGHZ: Vegetable Germplasm Bank of Zaragoza (Spain); CGN: Centre for Genetic Resources (Wageningen, Netherlands). ^b^ C: Control (full irrigation, week 1: 1350 mL, weeks 2–3: 2100 mL/each), DI-1 (Deficit Irrigation 1, week 1: 450 mL, weeks 2–3: 150 mL/each); DI-2 (Deficit Irrigation 2, weeks 1–3: 0 mL).

## Data Availability

Data generated during the study are within the article or supplementary material.

## Supplementary Materials

The following supporting information can be downloaded at: https://www.mdpi.com/article/10.3390/ijms24033052/s1.
